# Supplementary material for: A CRISPR-Cas9 delivery system for in vivo screening of genes in the immune system
Source: Nat Commun. 2019 Apr 10;10:1668. doi: 10.1038/s41467-019-09656-2 (PMC6458184; doi:10.1038/s41467-019-09656-2)
Supplement: Supplementary file 1 — Supplementary Information [file 41467_2019_9656_MOESM1_ESM.pdf]

A CRISPR-Cas9 delivery system for *in vivo* screening of genes in the immune system

LaFleur et al.

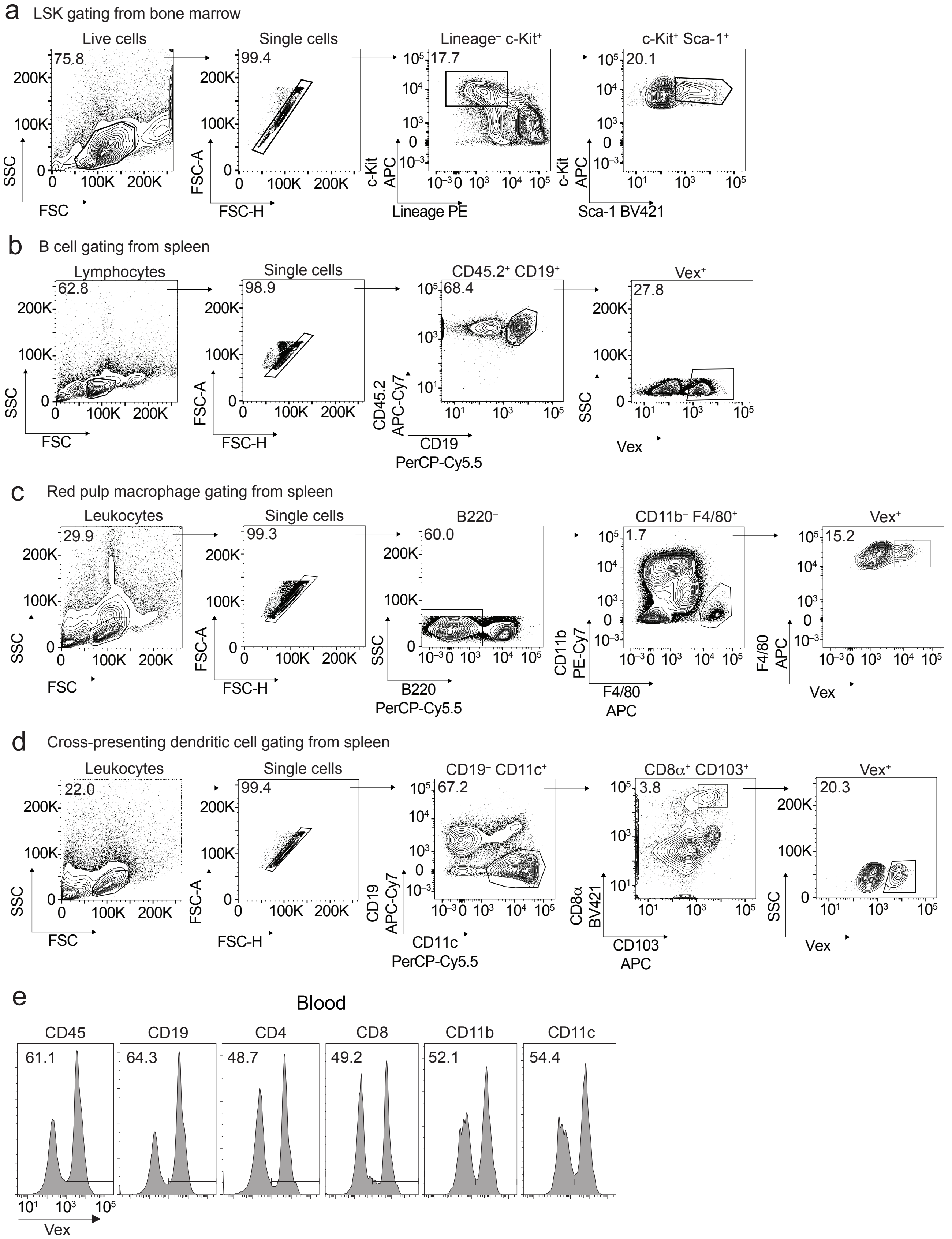

Supplementary Figure 1: Transduced immune cells are robustly recovered in CHIME-generated chimeras
(a) Representative flow cytometry plots for sorting of LSK cells (used for all chimera experiments). (b) Representative flow cytometry plots for gating of Vex⁺ CD19⁺ B cells for Figure 1b, Figure 1d, and Supplementary Figure 3d. (c) Representative flow cytometry plots for gating of Vex⁺ red-pulp macrophages for Figure 1b. (d) Representative flow cytometry plots for gating of Vex⁺ cross-presenting dendritic cells for Figure 1b. (e) Representative flow cytometry plots of Vex expression on major immune lineages in mice transduced with a non-targeting control sgRNA. All experiments had at least four biological replicate animals per group and are representative of two independent experiments.

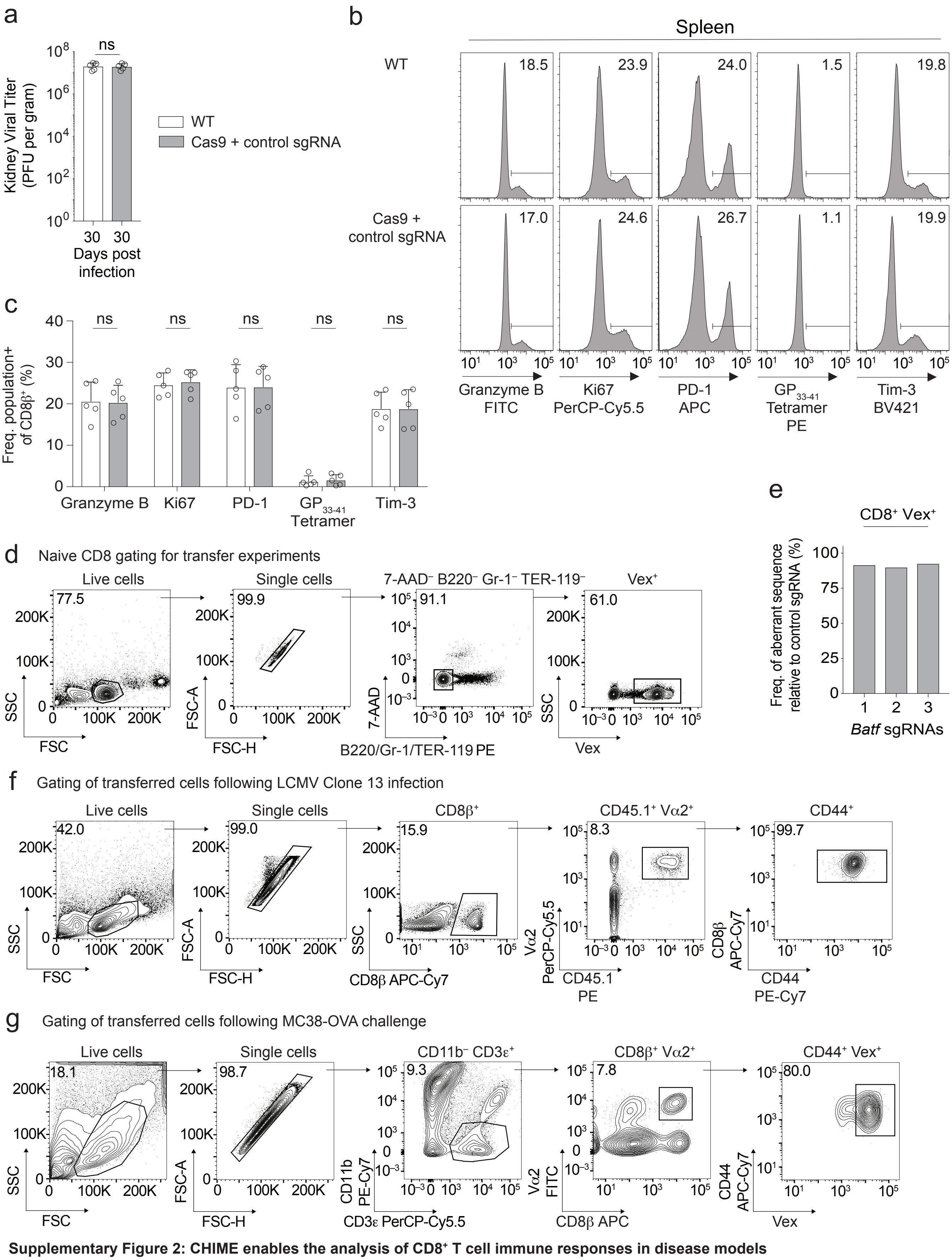

Supplementary Figure 2: CHIME enables the analysis of CD8⁺ T cell immune responses in disease models
(a) Day 30 kidney viral titer following LCMV Clone 13 infection of WT or Cas9 + sgRNA chimeras. (b) Representative flow cytometry plots of Granzyme B, Ki67, PD-1, GP_{33-41} tetramer, and Tim-3 expression on $CD8^+$ T cells at day 30 post LCMV Clone 13 infection. (c) Quantification of (b). (d) Representative flow cytometry plots for gating of Vex^+ naive $CD8^+$ T cells for transfer experiments for Figure 1d, Figures 2b-i, and transfer experiments in Figures 3-5. (e) TIDE assay on naïve $CD8^+$ T cells containing *Batf* sgRNAs prior to transfer into the competitive assay. (f) Gating strategy for analyzing transferred $CD8^+$ T cells from the spleen following LCMV Clone 13 infection for Figures 3b-c, Figures 4b-h, Supplementary Figures 3h-j, and Figure 5. (g) Gating strategy for analyzing transferred $CD8^+$ T cells from the tumor following tumor challenge for Figures 3d and 3e. All experiments had five biological replicate animals per group and are representative of two independent experiments. Bar graphs represent mean and error bars represent standard deviation. Statistical significance was assessed among the replicate bone marrow chimeras by two-sided unpaired t-test (a), or one-way ANOVA (c) (ns p>.05, * p<.05, ** p<.01, *** p<.001, **** p<.0001). Source data are provided as a source data file.

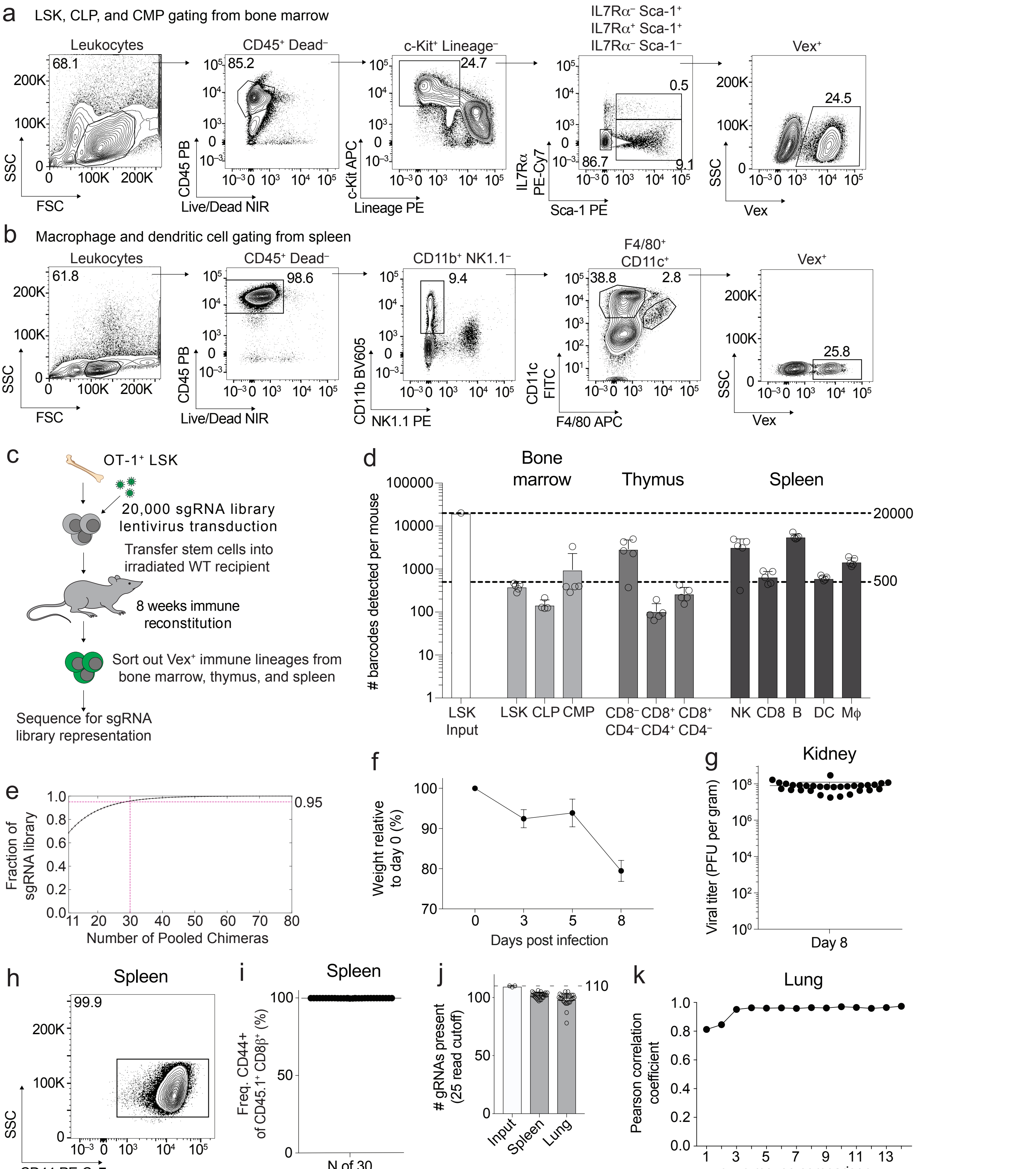

Supplementary Figure 3: Bandwidth experiments increase the potential scale of *in vivo* CHIME screening

(a) Representative flow cytometry plots for gating of Vex⁺ bone marrow subsets for Supplementary Figure 3d. (b) Representative flow cytometry plots for gating of Vex⁺ macrophages and dendritic cells from the spleen for Supplementary Figure 3d. (c) Schematic of bandwidth experiments. (d) Quantification of detected barcodes per mouse in the different immune populations analyzed using a 25 read cutoff. (e) Plot of Monte Carlo simulation results following pooling of 11-80 chimeras to recover a 5000 sgRNA library. Magenta dotted lines depict 30 chimeras (x-axis) and 0.95 (y-axis). (f) Weight loss of animals from LCMV Clone 13 screen normalized to day 0. (g) Kidney viral titer of animals from LCMV Clone 13 screen normalized to kidney weight. (h) Representative flow cytometry plots analyzing CD44 expression on $CD45.1^{+}$ $CD8^{+}$ T cells from the spleen of mice from LCMV Clone 13 screen. (i) Quantification of (h). (j) Quantification of sgRNAs detected with 25 read cutoff from experiment in LCMV Clone 13 screen. (k) Plot of Pearson R correlation vs. the number of replicate mice analyzed in the lung from mice used in experiment in LCMV Clone 13 screen. The bandwidth experiment had five biological replicate animals per group and is representative of one experiment. The *in vivo* screen had thirty biological replicate animals per group and is representative of one experiment. Bar graphs represent mean and error bars represent standard deviation. Source data are provided as a source data file.

Supplementary Table 1: STARS analysis of *in vivo* screen, Related to Figures 4d-h

Spleen Enriched

Gene Symbol	Number of perturbations	Ranks of perturbations	Perturbations	Most enriched perturbation	STARS Score	Average Score	p-value	FDR
Pdcd1	4	1;2;12;27	GCAATACAGGGATAC;GACACACGGCGCAAT;GCAGCTTGTCCAACT;GCACCCCAAGGCAAA	2	2.718512	2.23615	0.003794	0.075873
Kdr	4	6;10;11;63	GATACGTTTGAGAAC;GTTACCCGCTTAACG;GTCGGACTTGACTGC;GTTGTAAAGAATGGA	3	2.443697	1.383396	0.006477	0.06477
Adora2a	4	4;7;23;38	GGAGAGCCAACCCCT;GTGTCGATGGCAATA;GCTCCACAAAGACCT;GAAGCAGTTGATGAT	4	1.846436	1.485582	0.030627	0.204179
Ptpn2	4	9;16;41;44	GCACTCTATGAGGAT;GAATATGAGAAAGTA;GAAGAAGTTACATCT;GTGCAGTGATCCATT	4	1.59176	1.026825	0.058108	0.290539
Olfr5	3	21;22;65	GTGGGTCATACAAGC;GTACACTACCCAGTA;GACATGACAGGTTGA	2	1.017729	0.709741	0.211659	0.846634
Dgkz	4	20;37;42;95	GCAAGTCTTTGATCT;GACCCAGTGGTGTCT;GAGCACATCCGAGTA;GATGGAGCCACTGAC	3	0.861254	0.595126	0.297432	0.991441
Rnf128	4	36;39;43;70	GCAACTACACCAAGA;GATAATTACGGCAGC;GGGCAACCAGACGAT;GTCACAATGGTCATC	3	0.837031	0.631124	0.30997	0.885628
Socs3	4	3;31;46;77	GCGGATAAGAAAGGT;GTGAGCGTCAAGACC;GACTTCGGACGAGGG;GTTGAGTACACAGTC	3	0.769057	0.749086	0.357992	0.89498
Havcr2	4	30;33;100;104	GCTAAAGGGCGATCT;GTTACACTCTATCTA;GATCAGTTCTGAGCA;GTTCTCCAAGAACCC	2	0.57741	0.477265	0.571964	1
Cpt1a	4	55;59;75;79	GCACATTGTCGTGTA;GACGTTGGACGAATC;GCATACTGCTGTATC;GAGGAATACATCTAC	4	0.575062	0.500489	0.578395	1
Prkab2	4	13;73;83;94	GTACAAACTCTTTGT;GTCCTCACCTCTTTA;GAGGAGCACAAGATC;GTTCAGACCAGCGGA	2	0.52437	0.506811	0.672218	1
Lag3	4	15;72;86;102	GGGTGCGGGTTGTCT;GTCAGCAGCGTACAC;GATCCTAACTTTCTA;GAGAGAAGTCCCCGC	2	0.513187	0.483718	0.691696	1
Hk2	4	49;69;71;89	GGAGATGCGTCACAT;GAAGGCCATCCAGCG;GATCCGGAGTTGACC;GATTCCCGAGGACAT	2	0.484154	0.473383	0.754569	1
Il1r1	4	19;64;78;87	GCAGCAAGACCCCCA;GCGTATGTCCTATAC;GAGCATACAATTGTA;GACTGTGTTAGAGAA	2	0.449544	0.413085	0.852972	1
Tnfrsf9	4	28;58;68;85	GTTGCTCCTCTACCC;GCCAAGTACCTTCTC;GTGCATACGTACTTC;GACCAGGCTGACAGT	3	0.442735	0.415401	0.871108	1
Entpd1	4	60;62;80;84	GAGCTATCACAGCCA;GCTTGGATGTTGGTA;GCCAACCTGTACATC;GTGGATGAAGTCATC	2	0.440154	0.501908	0.885728	1
Tnfrsf1a	4	32;50;82;99	GGATCCCGTGCCTGT;GAGTTGCAAGACATG;GATGGGGATACATCC;GAGACCTAGCAAGAT	2	0.433177	0.396722	0.916216	1
Ifngr1	4	18;51;81;106	GTATGTGGAGCATAA;GTTCAGGGTGAAATA;GGTATTCCCAGCATA;GTATACCAATACGCA	2	0.430575	0.407703	0.930049	1
Tigit	4	40;53;74;107	GAGCCATGGTTCCTC;GTCTCTGACAATGAA;GCAGGCACGATAGAT;GCCTATCATACGTAT	2	0.427118	0.417596	0.956604	1
Olfr8	3	24;67;88	GAAGTACATGGGTGT;GATGTAGGATGCTAA;GTACAAGATTCATTA	2	0.361439	0.379644	0.990423	0.990423

Spleen Depleted

Gene Symbol	Number of perturbations	Ranks of perturbations	Perturbations	Most enriched perturbation	STARS Score	Average Score	p-value	FDR
Zap70	4	1;2;3;20	GTCTGGCGCGTACCA;GGAGATCCCTGTGAG;GTCGACAACCCCTAC;GAAGCGAGAGAATCT	3	4.102763	2.808489	4.63E-05	0.000879
Cd28	4	6;8;18;21	GTCGGCATTCGAGCG;GCTTGTGGTAGATAG;GTTCCTACAACCTTC;GCAAGGGCGTGAACA	4	2.876694	1.752224	0.002082	0.019778
Dlat	4	10;13;14;64	GACTACCGCAACGGA;GCGACAAGGCCACCA;GCAGGCTCTCAAACC;GTTCAGAACCACACC	3	2.142855	1.208302	0.014573	0.092297
Entpd1	4	27;31;49;51	GTGGATGAAGTCATC;GCCAACCTGTACATC;GCTTGGATGTTGGTA;GAGCTATCACAGCCA	4	1.33529	0.756809	0.103817	0.49313
Havcr2	4	7;11;78;81	GTTCTCCAAGAACCC;GATCAGTTCTGAGCA;GTTACACTCTATCTA;GCTAAAGGGCGATCT	2	1.313364	0.726743	0.11034	0.419292
Cpt1a	4	32;36;52;56	GAGGAATACATCTAC;GCATACTGCTGTATC;GACGTTGGACGAATC;GCACATTGTCGTGTA	4	1.172819	0.68583	0.15059	0.476868
Hk2	4	22;40;42;62	GATTCCCGAGGACAT;GATCCGGAGTTGACC;GAAGGCCATCCAGCG;GGAGATGCGTCACAT	4	0.996004	0.6845	0.217164	0.589445
Prkab2	4	17;28;38;98	GTTCAGACCAGCGGA;GAGGAGCACAAGATC;GTCCTCACCTCTTTA;GTACAAACTCTTTGT	3	0.966827	0.686639	0.237474	0.564001
Lag3	4	9;25;39;96	GAGAGAAGTCCCCGC;GATCCTAACTTTCTA;GTCAGCAGCGTACAC;GGGTGCGGGTTGTCT	3	0.939059	0.756021	0.256072	0.540597
Tigit	4	4;37;58;71	GCCTATCATACGTAT;GCAGGCACGATAGAT;GTCTCTGACAATGAA;GAGCCATGGTTCCTC	4	0.760537	0.681916	0.365348	0.694161
Il1r1	4	24;33;47;92	GACTGTGTTAGAGAA;GAGCATACAATTGTA;GCGTATGTCCTATAC;GCAGCAAGACCCCCA	3	0.747877	0.568364	0.377469	0.651993
Rnf128	4	41;68;72;75	GTCACAATGGTCATC;GGGCAACCAGACGAT;GATAATTACGGCAGC;GCAACTACACCAAGA	4	0.665326	0.496785	0.457599	0.724532
Tnfrsf1a	4	12;29;61;79	GAGACCTAGCAAGAT;GATGGGGATACATCC;GAGTTGCAAGACATG;GGATCCCGTGCCTGT	2	0.645653	0.562173	0.476752	0.696792
Tnfrsf9	4	26;43;53;83	GACCAGGCTGACAGT;GTGCATACGTACTTC;GCCAAGTACCTTCTC;GTTGCTCCTCTACCC	3	0.634808	0.492947	0.488318	0.662718
Ifngr1	4	5;30;60;93	GTATACCAATACGCA;GGTATTCCCAGCATA;GTTCAGGGTGAAATA;GTATGTGGAGCATAA	2	0.626997	0.652686	0.501457	0.635179
Olfr8	3	23;44;87	GTACAAGATTCATTA;GATGTAGGATGCTAA;GAAGTACATGGGTGT	2	0.540608	0.473449	0.630303	0.748485
Ptpn2	4	67;70;95;102	GTGCAGTGATCCATT;GAAGAAGTTACATCT;GAATATGAGAAAGTA;GCACTCTATGAGGAT	2	0.493103	0.665067	0.73796	0.824779
Dgkz	4	16;69;74;91	GATGGAGCCACTGAC;GAGCACATCCGAGTA;GACCCAGTGGTGTCT;GCAAGTCTTTGATCT	2	0.484154	0.441225	0.754569	0.796489
Socs3	4	34;65;80;108	GTTGAGTACACAGTC;GACTTCGGACGAGGG;GTGAGCGTCAAGACC;GCGGATAAGAAAGGT	2	0.455168	0.407293	0.833449	0.833449

Lung Enriched

Gene Symbol	Number of perturbations	Ranks of perturbations	Perturbations	Most enriched perturbation	STARS Score	Average Score	p-value	FDR
Pdcd1	4	1;2;11;18	GACACACGGCGCAAT;GCAATACAGGGATAC;GCACCCCAAGGCAAA;GCAGCTTGTCCAACT	4	3.144481	2.43948	0.001295	0.025908
Adora2a	4	3;4;26;41	GTGTCGATGGCAATA;GGAGAGCCAACCCCT;GCTCCACAAAGACCT;GAAGCAGTTGATGAT	2	2.132688	1.559918	0.016794	0.167939
Kdr	4	7;12;19;55	GATACGTTTGAGAAC;GTCGGACTTGACTGC;GTTACCCGCTTAACG;GTTGTAAAGAATGGA	3	1.768209	1.224709	0.039648	0.264323
Ptpn2	4	9;13;44;46	GCACTCTATGAGGAT;GAATATGAGAAAGTA;GAAGAAGTTACATCT;GTGCAGTGATCCATT	4	1.514539	1.02761	0.068749	0.343743
Socs3	4	6;29;30;77	GCGGATAAGAAAGGT;GACTTCGGACGAGGG;GTGAGCGTCAAGACC;GTTGAGTACACAGTC	3	1.229057	0.807144	0.133796	0.535184
Rnf128	4	37;38;42;66	GCAACTACACCAAGA;GATAATTACGGCAGC;GGGCAACCAGACGAT;GTCACAATGGTCATC	4	0.887395	0.666794	0.27592	0.919732
Dgkz	4	20;35;43;95	GCAAGTCTTTGATCT;GACCCAGTGGTGTCT;GAGCACATCCGAGTA;GATGGAGCCACTGAC	3	0.837031	0.595315	0.30997	0.885628
Olfr5	3	28;34;74	GTACACTACCCAGTA;GTGGGTCATACAAGC;GACATGACAGGTTGA	2	0.703285	0.53068	0.416933	1
Cpt1a	4	60;68;73;79	GCACATTGTCGTGTA;GAGGAATACATCTAC;GCATACTGCTGTATC;GACGTTGGACGAATC	4	0.575062	0.536189	0.578395	1
Entpd1	4	54;72;86;89	GCTTGGATGTTGGTA;GAGCTATCACAGCCA;GTGGATGAAGTCATC;GCCAACCTGTACATC	2	0.513187	0.54987	0.691696	1
Tnfrsf9	4	23;59;62;85	GTTGCTCCTCTACCC;GCCAAGTACCTTCTC;GTGCATACGTACTTC;GACCAGGCTGACAGT	3	0.505094	0.439632	0.702429	1
Havcr2	4	27;39;94;99	GCTAAAGGGCGATCT;GTTACACTCTATCTA;GTTCTCCAAGAACCC;GATCAGTTCTGAGCA	2	0.502774	0.438843	0.717511	1
Hk2	4	61;71;92;97	GGAGATGCGTCACAT;GAAGGCCATCCAGCG;GATTCCCGAGGACAT;GATCCGGAGTTGACC	2	0.502774	0.605183	0.717511	1
Prkab2	4	8;69;81;87	GTACAAACTCTTTGT;GTCCTCACCTCTTTA;GAGGAGCACAAGATC;GTTCAGACCAGCGGA	2	0.484154	0.498144	0.754569	1
Lag3	4	16;65;83;102	GGGTGCGGGTTGTCT;GTCAGCAGCGTACAC;GATCCTAACTTTCTA;GAGAGAAGTCCCCGC	2	0.455168	0.447587	0.833449	1
Ifngr1	4	15;45;76;98	GTATGTGGAGCATAA;GTTCAGGGTGAAATA;GGTATTCCCAGCATA;GTATACCAATACGCA	2	0.455168	0.433002	0.833449	1
Il1r1	4	17;64;84;91	GCAGCAAGACCCCCA;GCGTATGTCCTATAC;GAGCATACAATTGTA;GACTGTGTTAGAGAA	2	0.449544	0.438578	0.852972	1
Tnfrsf1a	4	22;47;70;96	GGATCCCGTGCCTGT;GAGTTGCAAGACATG;GATGGGGATACATCC;GAGACCTAGCAAGAT	2	0.444543	0.419446	0.866898	0.912524
Tigit	4	40;63;78;108	GAGCCATGGTTCCTC;GTCTCTGACAATGAA;GCAGGCACGATAGAT;GCCTATCATACGTAT	2	0.444543	0.417592	0.866898	0.912524
Olfr8	3	25;57;80	GAAGTACATGGGTGT;GATGTAGGATGCTAA;GTACAAGATTCATTA	3	0.414908	0.405406	0.977377	0.977377

Lung Depleted

Gene Symbol	Number of perturbations	Ranks of perturbations	Perturbations	Most enriched perturbation	STARS Score	Average Score	p-value	FDR
Zap70	4	1;2;4;10	GTCTGGCGCGTACCA;GGAGATCCCTGTGAG;GTCGACAACCCCTAC;GAAGCGAGAGAATCT	4	4.165571	3.016835	4.63E-05	0.000925
Cd28	4	5;7;23;29	GTCGGCATTCGAGCG;GCTTGTGGTAGATAG;GTTCCTACAACCTTC;GCAAGGGCGTGAACA	4	2.315979	1.581827	0.010502	0.10502
Dlat	4	6;8;18;75	GCGACAAGGCCACCA;GACTACCGCAACGGA;GCAGGCTCTCAAACC;GTTCAGAACCACACC	3	1.833905	1.199382	0.032339	0.215591
Hk2	4	14;19;40;50	GATCCGGAGTTGACC;GATTCCCGAGGACAT;GAAGGCCATCCAGCG;GGAGATGCGTCACAT	4	1.369691	0.91608	0.097062	0.485311
Cpt1a	4	32;38;43;51	GACGTTGGACGAATC;GCATACTGCTGTATC;GAGGAATACATCTAC;GCACATTGTCGTGTA	4	1.33529	0.766896	0.103817	0.415267
Entpd1	4	22;25;39;57	GCCAACCTGTACATC;GTGGATGAAGTCATC;GAGCTATCACAGCCA;GCTTGGATGTTGGTA	4	1.142071	0.800368	0.157391	0.524636
Havcr2	4	12;17;72;84	GATCAGTTCTGAGCA;GTTCTCCAAGAACCC;GTTACACTCTATCTA;GCTAAAGGGCGATCT	2	0.989556	0.637312	0.228082	0.651664
Prkab2	4	24;30;42;103	GTTCAGACCAGCGGA;GAGGAGCACAAGATC;GTCCTCACCTCTTTA;GTACAAACTCTTTGT	3	0.861254	0.622685	0.297432	0.743581
Lag3	4	9;28;46;95	GAGAGAAGTCCCCGC;GATCCTAACTTTCTA;GTCAGCAGCGTACAC;GGGTGCGGGTTGTCT	3	0.769057	0.676946	0.357992	0.795538
Tigit	4	3;33;48;71	GCCTATCATACGTAT;GCAGGCACGATAGAT;GTCTCTGACAATGAA;GAGCCATGGTTCCTC	4	0.760537	0.765895	0.365348	0.730696
Il1r1	4	20;27;47;94	GACTGTGTTAGAGAA;GAGCATACAATTGTA;GCGTATGTCCTATAC;GCAGCAAGACCCCCA	3	0.747877	0.611389	0.377469	0.686308
Rnf128	4	45;69;73;74	GTCACAATGGTCATC;GGGCAACCAGACGAT;GATAATTACGGCAGC;GCAACTACACCAAGA	4	0.688644	0.512424	0.422669	0.704449
Tnfrsf9	4	26;49;52;88	GACCAGGCTGACAGT;GTGCATACGTACTTC;GCCAAGTACCTTCTC;GTTGCTCCTCTACCC	3	0.652073	0.488047	0.463983	0.713821
Socs3	4	34;81;82;105	GTTGAGTACACAGTC;GTGAGCGTCAAGACC;GACTTCGGACGAGGG;GCGGATAAGAAAGGT	2	0.645653	0.470052	0.476752	0.681075
Ifngr1	4	13;35;66;96	GTATACCAATACGCA;GGTATTCCCAGCATA;GTTCAGGGTGAAATA;GTATGTGGAGCATAA	2	0.549161	0.499946	0.624705	0.83294
Tnfrsf1a	4	15;41;64;89	GAGACCTAGCAAGAT;GATGGGGATACATCC;GAGTTGCAAGACATG;GGATCCCGTGCCTGT	2	0.484154	0.473538	0.754569	0.943211
Dgkz	4	16;68;76;91	GATGGAGCCACTGAC;GAGCACATCCGAGTA;GACCCAGTGGTGTCT;GCAAGTCTTTGATCT	2	0.475903	0.435167	0.777007	0.914126
Ptpn2	4	65;67;98;102	GTGCAGTGATCCATT;GAAGAAGTTACATCT;GAATATGAGAAAGTA;GCACTCTATGAGGAT	2	0.468333	0.629646	0.796669	0.885188
Olfr8	3	31;54;86	GTACAAGATTCATTA;GATGTAGGATGCTAA;GAAGTACATGGGTGT	2	0.434081	0.397261	0.9034	0.950948
Olfr5	3	37;77;83	GACATGACAGGTTGA;GTGGGTCATACAAGC;GTACACTACCCAGTA	2	0.355561	0.353885	0.994078	0.994078

Supplementary Table 2: sgRNA and TIDE primer sequences			
Gene Target	sgRNA	TIDE Primer Forward	TIDE Primer Reverse
Pdcd1			
Pdcd1-1	GGTACCCTGGTCATTCACTT	CCCCACCTCTAGTTGCCTGTT	GGCATTTCACCTGTAAAACCCAC
Pdcd1-2	ACAGCCCAAGTGAATGACCA	CACCTCTAGTTGCCTGTTCTCCC	GGGGTGGATTTTGAGCCCCA
Pdcd1-3	GACACACGGCGCAATGACAG	GTACAGGCTCCTTCCTCACAGC	TCCATCCCTTAAAGGTAAATGGGCATC
Batf			
Batf-1	AGAGATCAAACAGCTCACCG	ATAGACAGCAATCAGCAGTTGCC	AAGGGATCACGGGAGTAGCAT
Batf-2	GTGGGTACTCACCAGGTGAA	AGGAGACCCAAGGGTGGGTA	TACATGCATGGGAGAGCGAAG
Batf-3	TGTGAAGTACTTGAGCTCCT	ATAGACAGCAATCAGCAGTTGCC	AAGGGATCACGGGAGTAGCATC
Ptpn2			
Ptpn2-1	GAATATGAGAAAGTATCGAA	GGGCACTGAGCAGCAAACTTTAT	GTGACTAGCTTTCATCTTTGCCTCTT
Ptpn2-2	CTCACTTCCATTATACCACC	CTGGAAGGCTGGCTGTAGTGTT	CTAACCTCCTCAGGCACCAGTC
Ly75			
Ly75-1	GTCACGAAACTCCATAATGG		
Ly75-2	GCTTGCTTGAGAAAACGTAA		
Ms4a1			
Ms4a1-1	GTCACGAAACTCCATAATGG		
Ms4a1-2	GCTTGCTTGAGAAAACGTAA		
Fcgr1			
Fcgr1-1	AGAGTACCATATAGCAAGGG		
Fcgr1-2	TGGGATGCTATAACTAGGCG		
Control			
Control-1	GCGAGGTATTCGGCTCCGCG		
Control-2	GCTTTCACGGAGGTTCGACG		

110 sgRNA Screen	
Entpd1-1	GTGGATGAAGTCATCACTGAG
Entpd1-2	GAGCTATCACAGCCAAGATAG
Entpd1-3	GCCAACCTGTACATCTACAAG
Entpd1-4	GCTTGGATGTTGGTATCAGTT
Adora2a-1	GTGTCGATGGCAATAGCCAAG
Adora2a-2	GCTCCACAAAGACCTGCGGCG
Adora2a-3	GGAGAGCCAACCCCTACCAG
Adora2a-4	GAAGCAGTTGATGATGTGCAG
Kdr-1	GTTACCCGCTTAACGGTCCGT
Kdr-2	GATACGTTTGAGAACCTCACG
Kdr-3	GTTGTAAAGAATGGAGACACG
Kdr-4	GTCGGACTTGACTGCCCACTG
Ifngr1-1	GTATGTGGAGCATAACCGGAG
Ifngr1-2	GGTATTCCCAGCATACGACA
Ifngr1-3	GTTCAGGGTGAAATACGAGGA
Ifngr1-4	GTATACCAATACGCAAATACC
Tnfrsf1a-1	GAGTTGCAAGACATGTCGGAA
Tnfrsf1a-2	GAGACCTAGCAAGATAACCAG
Tnfrsf1a-3	GATGGGGATACATCCATCAG
Tnfrsf1a-4	GGATCCCGTGCCTGTCAAAG
Il1r1-1	GAGCATACAATTGTAGCCGTG
Il1r1-2	GCAGCAAGACCCCCATATCAG
Il1r1-3	GCGTATGTCCTATACGTTCCG
Il1r1-4	GACTGTGTTAGAGAATGACCC
Socs3-1	GTTGAGTACACAGTCGAAGCG
Socs3-2	GTGAGCGTCAAGACCCAGTCG
Socs3-3	GACTTCGGACGAGGGTTCCGT
Socs3-4	GCGGATAAGAAAGGTGCCCGC
Ptpn2-1	GAAGAAGTTACATCTTAACAC
Ptpn2-2	GAATATGAGAAAGTATCGAA
Ptpn2-3	GCACTCTATGAGGATAGTCAT
Ptpn2-4	GTGCAGTGATCCATTGCAGTG
Rnf128-1	GATAATTACGGCAGCAACCGT
Rnf128-2	GCAACTACACCAAGACTGAA
Rnf128-3	GTCACAATGGTCATCGAAGTA
Rnf128-4	GGGCAACCAGACGATACAGA
Cpt1a-1	GCACATTGTCGTGTACCACAG
Cpt1a-2	GCATACTGCTGTATCGTCGCA
Cpt1a-3	GAGGAATACATCTACCTGCG

Cpt1a-4	GACGTTGGACGAATCGGAACA
Hk2-1	GATTCCCGAGGACATCATGCG
Hk2-2	GGAGATGCGTCACATTGACA
Hk2-3	GATCCGGAGTTGACCTCACAA
Hk2-4	GAAGGCCATCCAGCGCAGAG
Dlat-1	GACTACCGCAACGGACCGCAG
Dlat-2	GCAGGCTCTCAAACCCAACAG
Dlat-3	GCGACAAGGCCACCATAGGTG
Dlat-4	GTTCAGAACCACACCTACCGG
Prkab2-1	GTTCAGACCAGCGGATAACGG
Prkab2-2	GTACAAACTCTTTGTCCCCG
Prkab2-3	GTCCTCACCTCTTTATCAGA
Prkab2-4	GAGGAGCACAAGATCATGGTG
Lag3-1	GATCCTAACTTTCTACGAAG
Lag3-2	GAGAGAAGTCCCCGCGCTGG
Lag3-3	GTCAGCAGCGTACACTGTCAG
Lag3-4	GGGTGCGGGTTGTCTAGGCG
Havcr2-1	GTTACACTCTATCTACACCT
Havcr2-2	GTTCTCCAAGAACCCTAACCA
Havcr2-3	GCTAAAGGGCGATCTCAACAA
Havcr2-4	GATCAGTTCTGAGCAACTCGT
Cd28-1	GTCGGCATTCGAGCGAAACTG
Cd28-2	GCTTGTGGTAGATAGCAACG
Cd28-3	GCAAGGGCGTGAACAGCGACG
Cd28-4	GTTCCTACAACCTTCTCGCAA
Tnfrsf9-1	GTGCATACGTACTTCGTCCA
Tnfrsf9-2	GCCAAGTACCTTCTCCAGCAT
Tnfrsf9-3	GTTGCTCCTCTACCCACAACG
Tnfrsf9-4	GACCAGGCTGACAGTTATCAC
Zap70-1	GAAGCGAGAGAATCTCCTCG
Zap70-2	GTCGACAACCCCTACATCGTG
Zap70-3	GGAGATCCCTGTGAGCAATG
Zap70-4	GTCTGGCGCGTACCACTTCAG
Pdcd1-1	GCAATACAGGGATACCCACTA
Pdcd1-2	GACACACGGCGCAATGACAG
Pdcd1-3	GCACCCCAAGGCAAAAATCG
Pdcd1-4	GCAGCTTGTCCAACTGGTCGG
Dgkz-1	GACCCAGTGGTGTCTTACGA
Dgkz-2	GATGGAGCCACTGACCGGGT
Dgkz-3	GAGCACATCCGAGTAGTGGTG
Dgkz-4	GCAAGTCTTTGATCTGAGCCA
Tigit-1	GCCTATCATACGTATCCTGGT
Tigit-2	GCAGGCACGATAGATACAAAG
Tigit-3	GTCTCTGACAATGAATGACA
Tigit-4	GAGCCATGGTTCCTCCAAGCG
Olfr5-1	GTGGGTCATACAAGCTATGAA
Olfr5-2	GACATGACAGGTTGAGTAATG
Olfr5-3	GTACACTACCCAGTACTCATG
Olfr8-1	GTACAAGATTCATTATGAGG
Olfr8-2	GAAGTACATGGGTGTGTGCAG
Olfr8-3	GATGTAGGATGCTAACAACCC
BRDN0000737505	GAAAAAGTCCGCGATTACGTC
BRDN0000737693	GAAAACGGCTCGATCGGTGAT
BRDN0000737637	GAAAACGTAATTATACCGAGC
BRDN0000738185	GAAAATTGCACCTTCCCGGCC
BRDN0000737801	GAAACCCCCGCGCGGAGCGTC
BRDN0000737467	GAAACCTAGCGTAGATTCGGC
BRDN0000737848	GAAACGAGGCTGTTCGTACAC
BRDN0000737609	GAAACTCATACGTAGCGAATC
BRDN0000737434	GAAACTCCCGTGTCAACCGAT
BRDN0000738254	GAAAGACGTGCATTCAGCGAG
BRDN0000737777	GAACATGTTAAGTCGCGTTAT
BRDN0000737611	GAACCAGCATTTGACCGCGCT
BRDN0000737528	GAACCCCGGCTGTCATCGCCG
BRDN0000738228	GAACCCGCCGGAACAATCAGC
BRDN0000737727	GAACCGGCTGCGCGTTTGCAA
BRDN0000737483	GAACCGTACTGCGAGGAGCAT
BRDN0000737872	GAACCTCGTCTCATGTACGAA
BRDN0000737516	GAACGCCCCGGATTTCGTTGA
BRDN0000737844	GAACGGCTGCGCCCGCGGCAA
BRDN0000737412	GAACGGGCGCAATACCCTTTT

*BRDN = Broad GPP Non-targeting control sgRNAs
