# Supplementary material for: A CRISPR-Cas9 delivery system for in vivo screening of genes in the immune system
Source: Nat Commun. 2019 Apr 10;10:1668. doi: 10.1038/s41467-019-09656-2 (PMC6458184; doi:10.1038/s41467-019-09656-2)
Supplement: Supplementary file 2 — Reporting Summary [file 41467_2019_9656_MOESM2_ESM.pdf]

nature research

Corresponding author(s): Arlene H. Sharpe
W. Nicholas Haining

Last updated by author(s): 2019/03/15

Reporting Summary

Nature Research wishes to improve the reproducibility of the work that we publish. This form provides structure for consistency and transparency in reporting. For further information on Nature Research policies, see Authors & Referees and the Editorial Policy Checklist.

Statistics

For all statistical analyses, confirm that the following items are present in the figure legend, table legend, main text, or Methods section.

n/a	Confirmed	
☐	☒	The exact sample size (*n*) for each experimental group/condition, given as a discrete number and unit of measurement
☐	☒	A statement on whether measurements were taken from distinct samples or whether the same sample was measured repeatedly
☐	☒	The statistical test(s) used AND whether they are one- or two-sided<br>*Only common tests should be described solely by name; describe more complex techniques in the Methods section.*
☒	☐	A description of all covariates tested
☐	☒	A description of any assumptions or corrections, such as tests of normality and adjustment for multiple comparisons
☐	☒	A full description of the statistical parameters including central tendency (e.g. means) or other basic estimates (e.g. regression coefficient) AND variation (e.g. standard deviation) or associated estimates of uncertainty (e.g. confidence intervals)
☐	☒	For null hypothesis testing, the test statistic (e.g. *F*, *t*, *r*) with confidence intervals, effect sizes, degrees of freedom and *P* value noted<br>*Give P values as exact values whenever suitable.*
☒	☐	For Bayesian analysis, information on the choice of priors and Markov chain Monte Carlo settings
☒	☐	For hierarchical and complex designs, identification of the appropriate level for tests and full reporting of outcomes
☐	☒	Estimates of effect sizes (e.g. Cohen's *d*, Pearson's *r*), indicating how they were calculated

Our web collection on statistics for biologists contains articles on many of the points above.

Software and code

Policy information about availability of computer code

Data collection	Flow collection was performed using FACSDIVA (BD Pharmingen). Sequencing data was collected on a Hiseq (Illumina) or ABI3730xl DNA analyzer (Thermo Fisher Scientific).
Data analysis	All flow data was analyzed using FlowJo version 10.4.2. All statistical tests were run using Graphpad Prism 7. Sanger sequencing for the TIDE assay was analyzed on the online TIDE webtool. In vivo screen data was analyzed using STARS software and MATLAB.

For manuscripts utilizing custom algorithms or software that are central to the research but not yet described in published literature, software must be made available to editors/reviewers. We strongly encourage code deposition in a community repository (e.g. GitHub). See the Nature Research guidelines for submitting code & software for further information.

Data

Policy information about availability of data

All manuscripts must include a data availability statement. This statement should provide the following information, where applicable:

- Accession codes, unique identifiers, or web links for publicly available datasets
- A list of figures that have associated raw data
- A description of any restrictions on data availability

The data and materials that support the findings of this study are available from the corresponding author upon reasonable request. Source data underlying graphs in Figures 1-5 and Supplementary Figures 2 and 3 has been provided as a source data file.

Field-specific reporting

Please select the one below that is the best fit for your research. If you are not sure, read the appropriate sections before making your selection.

☒ Life sciences ☐ Behavioural & social sciences ☐ Ecological, evolutionary & environmental sciences

For a reference copy of the document with all sections, see nature.com/documents/nr-reporting-summary-flat.pdf

Life sciences study design

All studies must disclose on these points even when the disclosure is negative.

Sample size	Sample size was chosen to ensure the possibility of statistical analysis and to also minimize the use of animals in accordance with animal care guidelines from the Harvard Medical School Standing Committee on Animals and the National Institutes of Health. The results from previous results were also used to determine the sample size.
Data exclusions	Data exclusion was not used.
Replication	All attempts to reproduce our findings were successful.
Randomization	Age and sex-matched animals were used for each experiment. Animals were also co-housed when possible.
Blinding	Steady state analysis of the chimeras and the LCMV Clone 13 infection experiment (Figures 1d-f) were blinded during data collection. Co-transfer experiments are inherently blinded.

Reporting for specific materials, systems and methods

We require information from authors about some types of materials, experimental systems and methods used in many studies. Here, indicate whether each material, system or method listed is relevant to your study. If you are not sure if a list item applies to your research, read the appropriate section before selecting a response.

Materials & experimental systems

n/a	Involved in the study	
☐	☒	Antibodies
☐	☒	Eukaryotic cell lines
☒	☐	Palaeontology
☐	☒	Animals and other organisms
☒	☐	Human research participants
☒	☐	Clinical data

Methods

n/a	Involved in the study	
☒	☐	ChIP-seq
☐	☒	Flow cytometry
☒	☐	MRI-based neuroimaging

Antibodies

Antibodies used	Antibodies and dyes used include: 7-AAD BD Biosciences Cat# 559925 (1:100 dilution), Ki67-PerCP-Cy5.5 BD Biosciences Cat# 561284 (1:100 dilution), B220 Biolegend Cat# 103208, 103326 (1:100 dilution), CD11b Biolegend Cat# 101208, 101216 (1:100 dilution), CD11c Biolegend Cat# 117307, 117328 (1:100 dilution), CD127 Biolegend Cat# 135014, 135024 (1:100 dilution), CD19 Biolegend Cat# 115533 (1:100 dilution), CD20 Biolegend Cat# 150412 (1:100 dilution), CD25 Biolegend Cat# 101904 (1:100 dilution), CD3ε Biolegend Cat# 100220, 100308, 100336 (1:100 dilution), CD4 Biolegend Cat# 100516, 100531, 100543 (1:100 dilution), CD44 Biolegend Cat# 103008, 103028, 103030 (1:100 dilution), CD45.1 Biolegend Cat# 110708, 110716 (1:100 dilution), CD45.2 Biolegend Cat# 109824, 109832 (1:100 dilution), CD49b Biolegend Cat# 108909 (1:100 dilution), CD5 Biolegend Cat# 100608 (1:100 dilution), CD62L Biolegend Cat# 104417 (1:100 dilution), CD64 Biolegend Cat# 139303 (1:100 dilution), CD69 Biolegend Cat# 104513 (1:100 dilution), CD8α Biolegend Cat# 100737 (1:100 dilution), CD8β Biolegend Cat# 126606, 126608, 126610, 126620 (1:100 dilution), c-Kit Biolegend Cat# 135108 (1:100 dilution), F4/80 Biolegend Cat# 123116 (1:100 dilution), Gr-1 Biolegend Cat# 108408 (1:100 dilution), Granzyme B Biolegend Cat# 515403 (1:100 dilution), NK1.1 Biolegend Cat# 108708, 108732 (1:100 dilution), PD-1 Biolegend Cat# 135206, 135209 (1:100 dilution), Sca-1 Biolegend Cat# 108108, 108128 (1:100 dilution), TCR Vα2 Biolegend Cat# 127814 (1:100 dilution), TCR Vβ5 BD Biosciences Cat# 562087 (1:100 dilution), Ter-119 Biolegend Cat# 116208 (1:100 dilution), Tim-3 Biolegend Cat# 119703, 119723 (1:100 dilution), TruStain fcX Biolegend Cat# 101320 (1:50 dilution), Rat IgG2a κ Isotype Biolegend Cat# 400508 (1:100 dilution), Rat IgG2b κ Isotype Biolegend Cat# 400612 (1:100 dilution), and Near-IR Fixable Live/Dead Thermo Fisher Scientific Cat# L34976 (1:500 dilution). GP33-41 tetramer was obtained from the NIH Tetramer Core Facility and used at a 1:400 dilution. Cross-linking and depleting antibodies (CD3ε, CD28, CD4) were purchased from BioXCell.
Validation	Validation for mouse specificity was confirmed in the data sheets provided by the manufacturer.

Eukaryotic cell lines

Policy information about cell lines

Cell line source(s)	MC38-OVA cells were created in the Sharpe and Haining labs. BHK and Vero cells were a gift from E. John Wherry. 293x cells were a gift from Cigall Kadoch.
Authentication	MC38-OVA cells were validated based on expression of a selectable marker and exome sequencing.
Mycoplasma contamination	All cell lines were confirmed mycoplasma negative.
Commonly misidentified lines (See ICLAC register)	293x (clone of HEK). These cells were used in the production of lentivirus which was validated by titering.

Animals and other organisms

Policy information about studies involving animals; ARRIVE guidelines recommended for reporting animal research

Laboratory animals	Female/male C57BL/6J mice were purchased from The Jackson Laboratory and used at age 7-10 weeks. C57BL/6-Tg(Zp3-cre)1Gwh/J, C57BL/6-Tg(TcraTcrb)1100Mjb/J, and B6.SJL-Ptprca Pepcb/BoyJ mice were purchased from The Jackson Laboratory and bred to B6J.129(B6N)-Gt(ROSA) 26Sortm1(CAG-cas9*,-EGFP)Fezh/J mice (a gift from Feng Zhang). B6.Cg-Tcratm1Mom Tg(TcrLCMV)327Sdz mice were purchased from Taconic, backcrossed >10 generations to C57BL/6J from The Jackson Laboratory, and then bred to B6J.129(B6N)-Gt(ROSA) 26Sortm1(CAG-cas9*,-EGFP)Fezh/J mice.
Wild animals	N/A
Field-collected samples	N/A
Ethics oversight	HMA Standing Committee on Animals

Note that full information on the approval of the study protocol must also be provided in the manuscript.

Flow Cytometry

Plots

Confirm that:

☒ The axis labels state the marker and fluorochrome used (e.g. CD4-FITC).

☒ The axis scales are clearly visible. Include numbers along axes only for bottom left plot of group (a 'group' is an analysis of identical markers).

☒ All plots are contour plots with outliers or pseudocolor plots.

☒ A numerical value for number of cells or percentage (with statistics) is provided.

Methodology

Sample preparation	Bones were excised, mechanically crushed, filtered to single-cell suspension, ACK lysed, c-Kit MACS purified, and sorted. Spleen and lymph nodes were dissected from mice, mechanically minced, and filtered to single-cell suspension. RBC lysis was performed on spleen samples that were not MACS purified. For analysis of dendritic cells and macrophages from the spleen, samples were treated with collagenase for 20 minutes at 37°C prior to mechanical mincing. Livers were excised, mechanically minced, filtered to single-cell suspension, and enriched for lymphocytes on a Percoll gradient. Lungs were excised, mechanically minced, treated with collagenase for 20 minutes at 37°C, filtered to single-cell suspension, and enriched for lymphocytes on a Percoll gradient. Kidneys were excised and snap frozen in 1 mL RPMI 1% FBS media. Tumors were dissected from the surrounding fascia, mechanically minced, treated with collagenase for 20 minutes at 37°C, and filtered to single-cell suspension. Tumor-infiltrating leukocytes were enriched using a Percoll gradient. When necessary, cells were sorted on a FACS Aria II (BD Biosciences) to obtain greater than 95% purity.
Instrument	BD LSR II or BD Symphony A5 was used to collect data for analysis.<br>BD FACSAria IIu was used for cell sorting.
Software	All flow data was collected using FACSDIVA (BD Pharmingen) and analyzed using FlowJo version 10.4.2.
Cell population abundance	All sorts had a purity > 95%, checked by post-sort re-sampling.
Gating strategy	Gating strategy summarized in Supplementary Figures 1-3, with gates drawn based on single-stain and full-minus-one (FMO) controls.

☒ Tick this box to confirm that a figure exemplifying the gating strategy is provided in the Supplementary Information.
